# Detecting and understanding potential stigma among medical cannabis users in Germany

**DOI:** 10.1186/s12889-025-22084-w

**Published:** 2025-03-05

**Authors:** Velimir Borojevic, Felicitas Söhner

**Affiliations:** 1 Public Health Degree Program, Centre for Health and Society, Heinrich-Heine-University Düsseldorf, Prinzenpark Apotheke, Hansa-Allee 111, 40549 Düsseldorf, Germany; 2 Department of the History, Philosophy and Ethics of Medicine, Centre for Health and Society, Heinrich-Heine-University Düsseldorf, Moorenstr. 5, 40225 Düsseldorf, Germany

**Keywords:** Public health, Medical cannabis, Quality of life, Stigma, Disclosure

## Abstract

**Background:**

Despite its increasing prevalence among the public, cannabis use is still perceived as deviant behavior and consequently stigmatized. However, there remains a paucity of understanding regarding the impact of this stigma on patients employing cannabis for therapeutic purposes. This qualitative research endeavored to explore the stigma experiences of medical cannabis (MC) users in Germany, aiming to discern challenges that may impede their daily lives and healthcare access. The primary objective of this study was to identify instances of stigma associated with MC usage across various spheres.

**Methods:**

We conducted semistructured interviews with 15 individuals prescribed MC across diverse regions and occupational backgrounds in Germany. Interviews explored personal experiences with MC use, interactions with healthcare professionals, and stigma-related challenges. Data collection adhered to the COREQ guidelines. Transcribed interviews underwent systematic qualitative content analysis using MAXQDA software, with coding developed iteratively through researcher discussions. Communicative validation and inter-coder comparison enhanced analytical robustness.

**Results:**

Despite participants exhibiting a positive attitude towards the therapeutic effects and benefits of MC, stereotypes and prejudices persist. Participants highlighted the role of media portrayals and a lack of public awareness as central barriers to broader societal acceptance. Personal experiences with MC were marked by improved quality of life but also internalised stigma and external challenges, including interactions with law enforcement and difficulties with healthcare access.

**Conclusion:**

This qualitative study suggests that the utilisation of MC remains inadequately normalised in Germany. Our findings indicate that MC users experience both substantial benefits and persistent challenges, with stigma remaining a key issue. While participants reported improvements in quality of life, barriers such as bureaucratic hurdles and knowledge gaps among healthcare professionals hinder access to appropriate care. The findings underscore the imperative for enhanced education among healthcare professionals.

**Supplementary Information:**

The online version contains supplementary material available at 10.1186/s12889-025-22084-w.

## Introduction

### Historical and political background

In January 2017, Germany passed legislation (BT-DS 18/8965) allowing physicians to prescribe cannabis for medical purposes. Prior to this, patients had to apply for special exemptions from the Federal Opium Agency [1]. The “Cannabis as Medicine” law, enacted in March 2017, regulated the use of cannabis-based treatments, including dried flowers, and allowed patients to seek reimbursement from health insurers [2]. Prescriptions are granted primarily when no viable alternatives exist [3]. Despite this, one-third of prescription applications have been rejected annually, with concerns from patient and professional associations about the risk of driving patients back to the black market.

In 2024, Germany took steps toward legalising recreational cannabis, approving a law that will allow adults to possess up to 50 grams for personal use [4]. The evolving political landscape, alongside persistent barriers to medical access, raises questions about how MC users experience stigma, how it affects their daily lives, and its impact on access to care [5].

### Pharmacological effects of cannabis

Clinical evidence supports the use of cannabis for various conditions, including neuropathic pain of HIV-associated sensory neuropathy [6] or spasticity associated with multiple sclerosis [7]. MC treatment demonstrates both efficacy and tolerability in adult patients with Tourette Syndrome [8], and has shown promise in reducing the severity of self-reported headache and migraine [9].

Furthermore, MC has been found to alleviate pain, nausea and vomiting, and enhance food intake in cancer patients [10]. Preclinical research also suggests potential benefits for cancer patients, with documented anticarcinogenic effects across tumour types [11].

While recreational users often report experiencing euphoria after cannabis use, the extent to which MC can effectively treat anxiety, depression, and related disorders remains uncertain [12].

On the other hand, it is well-established that cannabis can also induce adverse effects, potentially leading to hazardous situations in the personal, professional, and health domains of its users. These effects may include hallucinations and memory impairment [13]. Empirical evidence has also highlighted psychosocial risks and mental health complications associated with frequent cannabis use, such as diminished educational attainment as well as potential harms from long term smoking as a preferred method of ingestion [14].

The discourse surrounding the impact of cannabis use on intellectual outcomes remains contentious. Certain studies suggest that long-term cannabis users may exhibit cognitive deficits [15]. It is limiting to emphasise that while there is increasing evidence for cannabis use in certain conditions (e.g. neuropathic pain, Tourette’s syndrome in adults), this evidence remains limited and disease-specific. Studies describing patterns of medicinal cannabis use in older adults emphasise the need for research to determine appropriate indications, precise drug doses and short- and long-term outcomes in older adults. However, it is important to note that there are still gaps in current knowledge on this topic and therefore the need for further research is high [16].

### Stigma and Cannabis

Stigma, as defined by sociologist Erving Goffman, refers to the exclusion of individuals from full social acceptance [17, 18]. According to Link and Phelan, the practice of labelling human differences and grouping people into categories can lead to social isolation and discrimination [19–21]. For MC users, stigma is a significant issue, particularly for those with mental health conditions [22, 23]. It can act as a barrier to accessing MC treatment, with patients often facing discrimination at both interpersonal and institutional levels [24]. While cannabis may offer a valuable alternative to opioids for many, stigma, along with the lack of clear guidance from healthcare providers, remains a challenge [25]. Despite increasing public acceptance of cannabis consumption in certain countries, individuals who use cannabis continue to encounter considerable stigma both at the interpersonal and institutional levels [26, 27].

Although to a lesser degree than other illicit substances, cannabis use tends to be stigmatized globally [28]. This stigma stems from the association of cannabis with crime and violence [29], and some MC users have reported experiencing stigma due to negative perceptions of cannabis as a recreational drug [26]. Although cannabis is increasingly accepted in some places, in Germany it occupies a “medical liminal space,” where its ambiguous legal status and ongoing debate about its risks and benefits leave users vulnerable to stigma [30]. It is noteworthy that not all cannabis users experience stigma to the same extent, and the degree of stigmatization varies among individuals [31, 32].

### State of the Art

While stigma associated with recreational cannabis use has been widely studied, research on how MC users experience and navigate stigma remains limited, particularly in Germany [33–37]. Qualitative studies from countries such as Canada, the USA, and Israel suggest that stigma shapes how MC users are perceived and treated, yet no such research has been conducted in the German context [26, 30, 38, 39].

Therefore, it is premature to assert whether stigma associated with cannabis has entirely dissipated, as there is insufficient evidence to support such a claim. One contributing factor may be the tendency to prioritize quantitative data over the nuanced narratives unearthed by qualitative research [29].

MC users operate within a complex social and legal landscape, where they may be seen as patients, clients, or even criminals [27].

Hence, adopting a local and culturally sensitive perspective is crucial in cannabis research, as demonstrated by certain international studies [34]. Stereotypes surrounding cannabis use, including associations with addiction, contribute to their marginalisation [40]. However, it is crucial to distinguish between recreational cannabis use and MC use, as patients often distance themselves personally from recreational cannabis consumers [39].

In Germany, the prohibitionist legacy has influenced healthcare attitudes, often framing MC patients as “difficult” [41]. Despite growing acceptance, stigma remains a potential barrier to care, underscoring the need for research that captures the lived experiences of MC users.

### Aim of the Work

The aim of the present study is to gain insight into the experiences of MC users in Germany, assessing the extent to which stigma impacts their daily lives and healthcare experiences. The interviews were designed to explore individual patients’ perceptions regarding their interactions with healthcare providers, while also gauging whether there is a need for increased public and professional awareness. Understanding how MC patients perceive, experience, and cope with stigma is essential for ensuring appropriate medical care for these individuals.

This study represents an initial exploration of the evolving medical landscape from the standpoint of MC patients in Germany. It examines how MC users perceive, experience, and navigate stigma, and its influence on their interactions with others and the healthcare system. To address these objectives, 15 semi-structured interviews were conducted with MC patients. Informed consent to participate in the study was obtained from all participants. The interviews used for this study have already been published elsewhere [42]. The purpose of the research was explained as part of the informed consent process. Details on the consent process are presented in the Additional file 2.

## Material and Methods

### Study design

Using semistructured audio interviews with individuals who are prescribed MC, this study aimed to gain an in-depth understanding of the examined social phenomena. Our study was deemed exempt by the Ethical Review Board of the University Hospital Düsseldorf (Identifier: 2022-2002). There was no participation incentive. The study adhered to the Consolidated Criteria for Reporting Qualitative Research (COREQ) guidelines [43].

Our study design was grounded in a qualitative and inductive-deductive research approach used in Oral History [44–46]. According to du Bois-Reymond, oral history research with a hermeneutic epistemological interest is never fully concluded, as each statement generates new questions and hypotheses[47]. To address this inherently open research situation, our study aimed for theoretical-empirical saturation by treating each interview as a case that provided analytical insights or raised new questions, guiding subsequent sampling and inquiry. This approach of theoretical sampling [45] enabled both substantive replication and differentiation: additional similar cases and contrasting cases were sought to clarify and refine emerging conceptual and thematic patterns. Inferential saturation was reached when interviews no longer yielded new insights [48].

### Study population - Recruitment and inclusion criteria

We conducted a targeted sampling approach to recruit a diverse range of MC users across different age groups, regions, and occupational fields (crafts, management, administration, and sports) who use MC for various medical conditions. Recruitment was carried out through the research team’s existing contacts in primary care, informational flyers, recommendations from the German Association of Cannabis Patients (Bund Deutscher Cannabis-Patienten e.V.^1^), and participant referrals for additional potential respondents. To assess the adequacy of the sample size, we relied on the information power criteria proposed by Malterud et al. [49], who introduced the concept of “information power” to determine an appropriate sample size for qualitative studies. According to this concept, the more relevant information a sample contains for the study, the fewer participants are required (Additional file 2).

The inclusion criteria for this study were as follows:

- *Age at least 18 years (legal age of majority)*
- *MC use, or receiving MC prescribed by a physician.*
- *Ability to give consent*
- *Ability to communicate on their own*

Between September and November 2022, 15 participants (5 women and 10 men) were recruted with an average age of 39 (range = 22-58, SD=9.199); no persons were excluded from the survey because they did not meet the inclusion criteria; no persons were excluded because they did not provide consent. All participants reported using cannabis in dried flower form, except for one individual who was using Dronabinol. An overview of the demographic and clinical characteristics of the study participants can be found in the table 1 below.

**Table 1:** Study population demographic and clinical characteristics

**Code**	**Age/Gender**	**Region**	**Diagnosis**	**Profession**
**P1**	22/F	NRW^a^	Rheumatism	Master Craftsman Exam
**P2**	45/F	NRW	Cancer	Working(unknown)
**P3**	38/M	NRW	GTS^b^	Unknown
**P4**	29/M	Hamburg	GTS^b^	Real estate manager
**P5**	45/M	NRW	Ulcerative colitis	Working with children/Sport
**P6**	26/M	NRW	Multiple sclerosis	Student
**P7**	42/M	NRW	Depression / ADHD^c^	Early retiree
**P8**	35/M	Niedersachsen	Pain Chronic Inflammation	Activist
**P9**	46/F	NRW	Cancer	Police officer
**P10**	29/F	NRW	Arthrosis	Pharmacy student
**P11**	49/M	NRW	ADHD^c^/ Physical disorder	Unknown
**P12**	40/F	Sachsen	Post-traumatic syndrome	Unknown
**P13**	39/M	NRW	Pain	Unknown
**P14**	58/M	NRW	Depression	Working(unknown)
**P15**	37/M	NRW	Chronic pain	Independent

^a^*NRW* North-Rhein-Westphalia

^b^*GTS* Gilles de la Tourette Syndrome

^c^*ADHD* Attention deficit hyperactivity disorder

All participants were provided with information stating that non-participation in the interview would have no consequences, and that participation was voluntary and could be withdrawn at any point during the study.

Information about the study was communicated directly to participants at the dispensary or sent via email. As all data were collected in Germany, the recruitment materials were in German and were not translated into English.

Qualitative research seeks to identify collective, shared, and divergent structures of meaning from various perspectives. Therefore, the objective of qualitative research is not statistical sampling aimed at generating representativeness through a large number of individuals, but rather theoretical sampling, in which typical cases are selected based on the research question [50].

### Interviews

One of the authors (V.B.), a public health scientist, conducted 15 interviews (range, 12-45 min; median, 21 min) following training and continuous evaluation by the qualitative research expert (F.S.) supervised by the science historian Chantal Marazia (C.M.). Participants were asked to describe their personal experiences with MC, the reactions they encountered from their social environment, and any experiences of stigmatisation. Previous research suggests that MC users face a significant risk of stigma from various sources, particularly when their diagnosis involves certain health conditions, such as mental illness [27, 28]. We followed the recommendations of the Oral History Association [51] to develop interview questions and our methodological approach, allowing for continuous adaptation and improvement of interview quality. The recruitment script, consent script, and interview guide are provided in (Additional file 2).

A comprehensive pre-test via videoconference, which included a technical test, was conducted prior to the main interviews. The audio interviews were recorded using Voice Memos on a Notebook. The choice of tool was determined by technical considerations and the preferences of the interviewees. With the exception of one interview, all were conducted online. The remaining interview took place in a quiet room at a pharmacy store, after an appointment was scheduled with the study participant. Conducting interviews online can alleviate organizational challenges such as scheduling, geographical distance, or travel expenses [52], and also enables the study to proceed independently of the constraints imposed by Covid-19.

The interviews were audio-recorded and transcribed using *Amberscript,* which transcribes the text exactly as it sounds including speech errors, false starts, filler words, slang words, repetitions, and stutters. Following transcription, corrections were made by one of the authors [V.B.], while retaining some repetitions and slang terms deemed pertinent for subsequent analysis.

The interviews were structured into three parts. The first part, the introduction, aims to establish a comfortable atmosphere and facilitate a seamless transition into the topic. The actual interview commenced with a brief introduction and a series of simple questions designed to “break the ice”. While these questions were easy for the interviewee to respond to, they were not mere small talk; rather, they were formulated to provide fundamental information relevant to the topic at hand. Gender and age data of the respondents were also recorded to enable comparison of important socio-demographic variables across groups. During this segment of the interview, the purpose and rationale behind the interview were carefully explained, prioritizing maximum data security. The main interview was recorded for analysis purposes. This introductory segment offers an initial general insight into the respondent’s perspective on various aspects, including the decision to use MC, interactions with healthcare professionals, daily experiences, and potential challenges encountered.

Guidelines for this semi-structured interview were prepared and formulated, though spontaneous questions were also welcomed. This section aimed to delve deeper into the patient’s desires, emotions, thoughts, and experiences related to potential stigmatization. Various facets of stigma, such as stereotypes, prejudice, and discrimination, were explored. Notably, questions about stigmatization were not directly posed, and efforts were made to avoid using the term “stigma”. As Obertreis suggests, “The more directly we ask, the more the silence about this part of the story sets in.” [53, 54]. The questions focused on general experiences and opinions, with a concerted effort to maintain objectivity.

In the third and final segment of the interview, participants were given the chance to express their thoughts on the overall situation and suggest areas for improvement. Additionally, they had the opportunity to provide feedback on the entire interview process (Additional file 2).

### Data analysis

We analysed systematicaly the fully transcribed interviews, originally recorded as audio files, to identify relevant themes and select representative excerpts essential for data interpretation.

The analytical process followed the principles of qualitative content analysis [55]. Given that qualitative research focuses on typification, theorisation, and the purposeful selection of cases rather than statistical probability sampling, theoretical sampling was employed [46]. This approach ensured that data collection was guided by theoretical considerations, allowing for the refinement and further development of emerging conceptual patterns [44, 56].

We began with an all-encompassing data table that was organised according to respondents and interview questions. The data was discussed by the researchers, reduced by increasingly concise representations and at the same time continuously supplemented by additional aspects. Qualitative data management software MAXQDA Analytics Pro 2022 was utilized to organize the data and conduct in-depth analysis. V.B. created a category system that focussed on the main codes that had emerged from the discussion of the previous table. The researchers (V.B. and F.S.) maintained regular contact to discuss and resolve discrepancies in the process of categorisation and qualitative content analysis.

To preserve anonymity while allowing for identification of study participants based on demographic factors such as age and sex, each quotation was attributed with a unique identifier corresponding to the interviewee. For instance, a male participant aged 38 would be represented as P3M, 38.

Transcripts were meticulously reviewed and analysed multiple times to gain a comprehensive understanding of the data. Using an inductive-deductive approach, sections highlighting emerging themes and ideas were identified and highlighted. Within respondents’ statements, smaller units of meaning pertaining to specific questions were identified, and pertinent information was extracted and condensed.

To ensure the validity of the results, communicative validation was employed. This involved discussing the categories, coding, and interpretations within the research team. Additionally, where necessary, the results were reviewed and discussed with the respondents themselves.

Given the subjective nature of interpretative elements, inter-coder comparison played a crucial role in enhancing the credibility of the analysis. Discussing the coding system among different researchers, in addition to the primary analyst, and striving for consensus among team members contributed to the overall credibility of the findings [57]. The categorisation system was repeatedly discussed via email by the researchers (V.B., F.S., C.M.) and in the Düsseldorf Oral History research group (Düsseldorfer Forschungswerkstatt Oral History^2^), leading to further refinement of the differences and commonalities within the concepts and themes.

Since the coding process was conducted in German, and the interviews were conducted in German as well, the decision was made not to translate the entire interviews into English to avoid the potential loss of information during translation. However, citations were translated into English for improved readability and flow, while efforts were made to maintain the original content of the citations through a 1:1 translation, word-for-word. The original German versions of each citation are provided in the Additional file 1.

## Results

In this part, four primary codes are outlined, providing insight into the overarching category of experiences with MC. Given the complexity of the field investigated, it is challenging to delineate these codes distinctly, as the experiences—whether personal, societal, involving family members, or healthcare professionals—are often intertwined.

The following table 2 on the category system was created by V.B. during the analysis process and regularly discussed by the researchers. Table 2 based on the coding tree shows the four main categories of the qualitative analysis, which are presented below. The table presents a summary of the key aspects expressed by the interviewees, organised into four main categories of qualitative analysis, which are outlined below.

**Table 2:** Category and codes

**Category**	***Experience with Medical Cannabis***			
**Codes**	Society	Family and Friends	Personal experience	Medical (Health) Professionals
**Subcodes**				
**POSITIVE**	-Better than before	-Support -Acceptance	-Power of the Cannabis -Quality of life	-to be lucky -Understanding
**NEGATIVE**	-Discrimination -Prejudice/Judgment -Ignorance -Stereotype -Isolation -Negative picture in media	-Contact loss -Changed relationships	-Self-stigma -Experience with police	-Disparity -Lack of knowledge - Exclusion (*we don´t want to deal with it)* -Prejudice
**NEUTRAL / IN BETWEEN**		-Accepted, but not 100% OK -Not everyone needs to know	-Not a Cannabis Fan, but it helps me -Health insurance and bureaucracy	-Unexperienced, but ready to help

### Society

This section examines the perceptions and attitudes of study participants regarding societal responses to MC use, excluding those from family, close friends, and medical professionals. While nearly all participants shared their personal experiences, only a minority reflected on broader societal reactions.

A prevailing theme among participants was the persistence of stigma, with many highlighting generational differences in attitudes. Several interviewees noted that older generations often perceive cannabis purely as a recreational drug and associate its use with social harm. In this study, participants were not asked whether they had used cannabis recreationally. However, a few voluntarily mentioned that they had tried cannabis before receiving it as a prescribed medication. All participants used MC for a specific illness under a doctor’s prescription.

Instances of discrimination and exclusion were frequently recounted. One participant described restrictions placed upon them in the workplace:

“At work they told me: No, you can’t take your medicine here and please don’t take it before you go on duty, because they said that you are under the influence of drugs and not focused” (p6).^3^

Historical context was also cited as a factor shaping contemporary societal views. One participant referenced the prohibition era as instrumental in fostering negative stereotypes:

“Cannabis users are not dangerous, but someone who uses cannabis is a cannabis user and not a serious criminal. Yes, in society this image should also be reconsidered, that these stereotypes were made by people who wanted to denounce others. I don’t need to explain the story of prohibition to you. You certainly know yourself that this has racist and economic backgrounds” (p12).

Several participants pointed to deeply ingrained prejudices within their communities. One interviewee reflected on being socialised into a negative perception of cannabis use:

“I think even if you have not had any contact with cannabis at some point in your life, then you have grown up very conservatively. Then, is the gateway drug, so to speak. And that’s what I was told when I was a teenager, in my case, by my parents” (p5).

Educational and professional stereotypes were also highlighted. One participant challenged the widespread assumption that MC users lack academic achievement:

“[…] Even people with higher education, because it is always assumed that these who smoke pot are only the losers in education, which of course is not true at all” (p9).

The enduring influence of the “stoner” stereotype was noted by multiple participants, with some asserting that, despite increasing acceptance, negative imagery remains pervasive:

“First of all, because of course this stoner image, which has been painted for decades, is still quite present” (p4).

Media portrayals were also mentioned as a contributing factor to societal perceptions of cannabis. One participant criticised the persistent negative framing of cannabis use in news coverage:

“Because you see it again and again in media reports, even when it comes to medical cannabis, that there is ALWAYS a super negative undertone [to put it very clearly]. Yes, you should be critical, you should. But you should be critical with every medication, with all opiates. You should be critical” (p1).

Distinctions between medical and recreational cannabis use were frequently emphasised. Several participants noted that they used MC to manage health conditions, yet society often fails to differentiate between medical patients and recreational consumers:

“Um, to make it short Bob Marley equals Bob Marley T-Shirt, dreadlocks and somehow a bag[joint]. And this is the bad thing that somehow many patients also add up to this cliché... ” (p11).

Lack of knowledge was identified as a key driver of negative societal perceptions. Participants underscored the need for greater public awareness and education. A police officer among the interviewees elaborated:

“[...] that they are also normal people who have hobbies, who have a family and whose hobbies are not related to cannabis from morning to night, but who just live a normal life and who should be let live. And I think that’s what people will then learn” (p9).

The role of societal recognition was further discussed, with one participant suggesting that deeply rooted beliefs, rather than scientific evidence, often dictate public attitudes towards MC. One participant, aged 58, elucidated his perspective on this matter:

“I think you can also sometimes assess patients a little bit in terms of whether this is a patient who is now very open to, let´s say pharmaceuticals, psychotropic drugs, these classic things that are also used very successfully. I don’t want to make it look bad or diverting attention away [from alternative approaches]. That’s also the idea of homeopathy, things like that. Yes, maybe you have to believe in it. There is no scientific evidence. And yet there are very many people who say it helps them [….] We have six children and I don’t want anything to happen about it, because it is not yet recognized in society. My wife is also a cannabis patient and we are suddenly called a ‘stoner family’. What’s going on with them? We’ll have to take a look. Things like that [meant: it is concerning and necessitates a closer examination of such matter] ” (p14).

Family and Friends

Most participants emphasised the importance of support from family and close friends, with the majority reporting strong backing from their immediate social circles. However, a few participants described negative experiences, ranging from strained relationships to complete loss of contact.

One participant recounted how their brother severed ties due to their status as an MC patient:

“[...] because my brother has completely broken off contact/stopped communicating with me because of this. So now I had nothing to do with him for four years [....] he studies administration of justice[...] he meant, yes, as a cannabis patient I have a bad influence on him and I am quite dangerous for his profession. (But), because as a judicial officer he is not allowed to do such things and should not have anything to do with such people. That is really unbelievable” (p10).

Another participant described how their family initially struggled to accept their MC use, despite eventually recognising its legitimacy. Nevertheless, lingering reservations remained, with relatives encouraging alternative treatments:

“My family not at all. So, I’ve always left out completely at family celebrations. My parents eventually accepted it, because I got the costs covered, but they kept telling me to stop, you’ll find something better. Another way [alternative treatment] to deal with the disease. And every time I went to visit my parents, I didn’t take my medicine because [...] I would have had to go off the property every time to be able to take it. And which actually then puts me back in a position where I’m at risk again, because I’m in the public domain” (p6).

Even among those who received support, some participants noted that they preferred to keep their MC use private, sharing it only with those they considered trustworthy:

“Whereby openly dealing with it doesn`t mean that everyone knows. Well, if somebody knows, it`s our closest relatives and friends, from whom we assume that they are also, well, let`s say, trustworthy enough” (p14).

For some, discretion appeared to be a conscious strategy to avoid social scrutiny, as one participant succinctly stated:

“[…] we don’t have to shout it from the rooftops” (p2).

### Personal-experience (Self-experience)

Participants widely emphasised the effectiveness of MC, noting its significant impact on their quality of life. While all suffered from chronic conditions, many expressed that MC allowed them to regain a sense of normality. A female cancer patient described how cannabis therapy helped her reclaim everyday activities:

“So, I’m now convinced about the subject of cannabis[...] would never have thought, I would be anyway, I would have, you don’t see it on me, I don’t have red eyes, nor that I somehow think ‘the world is so beautiful’. No, it makes me spring into action. I can, it(cannabis) can do something. I can go out with friends. It gives a quality of life back! [...] that this plant is greatly underestimated [....] I have gained nine kilo(grams) because of it, I am able to go back to work, I’m partially employed [managed to go back to work on a. partial basis]” (p2).

Others echoed similar sentiments, stating that MC had brought tangible improvements to their health. Despite this, not all participants identified as cannabis users in the traditional sense. While some embraced their status as MC patients, others would prefer an alternative treatment if it provided the same relief:

“To tell you honestly, quite frankly, If I did not have to take it from tomorrow on, I would be the happiest person in the world” (p3).

Some participants also reported struggling with internalised stigma. Feelings of fear, shame, and self-doubt were particularly prevalent in the early stages of MC use, with individuals questioning whether they should take their prescribed medication at all:

“The sad thing is that sometimes I myself still have this stigma in my head and then tend to abstain from medication in a situation where it is actually appropriate in order to avoid possible conflicts[...] You are directly labelled as someone of a category where you actually don’t belong and then they can’t believe that either” (p7)

For some, self-stigma was deeply personal, shaped by past experiences with addiction in their families:

“When I did not get it on medical prescription, I stopped several times. I was always afraid that I would be addicted. Okay, that was because of my father’s alcohol addiction. That’s where I am. As far as addiction is concerned, I was pretty warned and that’s why I always stigmatized myself.” (p11)

Beyond internal struggles, participants also faced external challenges, particularly in interactions with law enforcement. Some expressed frustration that police officers often lacked sufficient knowledge to differentiate between medical and recreational cannabis use:

“[...] is simply that I think that particularly for example police officers or similar I think should be better trained on medical cannabis” (p1).

Difficulties in obtaining health insurance coverage were another major concern. Many participants described the process of securing reimbursement as complex and, at times, dehumanising:

“[...] the health insurance company has to, because it is a drug available on prescription, simply pay for it” (p12).

“The medical service of the health insurance is partly already a very big obstacle for people to become cannabis patients… ” (p6).

### Medical professionals

The relationship between patients and their doctors was a subject of strong emotions among participants, ranging from gratitude to frustration. While some were fortunate to find doctors willing to prescribe MC, others encountered significant barriers. Several participants described long waits, outright rejections, or reluctance from medical professionals to engage with cannabis therapy. One participant reflected on their difficulty in being treated as a “normal” patient:

“Ultimately, if you say somewhere front ´I am cannabis patient´, the person´s gaze already changes. I noticed that in the hospital as well. I was in the hospital last year with a stomach ulcer. I had such stomach pain and when they heard cannabis, they didn’t want to examine me properly anymore ” (p3).

Many participants pointed to a general lack of knowledge about MC among healthcare professionals. Some doctors, they reported, dismissed cannabis therapy outright without consideration:

“But I still have... also often the feeling that many doctors simply reject it” (p1).

“And that is also where the main need for education lies, because there are very very few doctors open to cannabis therapy” (p8).

The frustration caused by such experiences was evident. One participant, who has been prescribed MC since 2017, highlighted the challenge of overcoming preconceived notions within the medical system:

“Yes, you’re always a bit, how should I say it, either the Guinea pig or the stupid stoner on the other side. Okay, so that has a lot to do with this pigeonhole that I was talking about earlier. It’s very difficult for people to make it. That’s why so many people deal with cannabis abuse, that is what you find on paper of some ADHD patient, just because the doctors directly pigeonhole the individual, although that was just a medical self-experiment. That is really difficult and really annoying for people who are not as I am” (p11).

Almost all participants agreed that greater investment in cannabis research is needed and that medical professionals should be better informed about its therapeutic potential. While a lack of experience among doctors was acknowledged, many participants felt that willingness to learn and refer patients to more knowledgeable colleagues could significantly improve access to treatment.

## Discussion

In this paper the experiences of MC users were explored ranging from positive to negative, and indicators of stigma were identified. A further aim was to comprehend the contexts in which participants encountered stigmatization and to understand the reasons behind it. The results are discussed below.

The participants in the study here discussed primarily grappled with chronic health problems, with two participants diagnosed with cancer reporting long-term beneficial effects of MC. The mentioned effects included improvements in wealth, energy levels, weight gain, and reductions in pain, vomiting, and nausea. During the interviews, the improvement of life quality emerged as a recurring theme. Many participants in the present study reported experiencing positive effects and an enhanced quality of life across various domains such as mental health, financial stability, employment, and recreational activities. The findings of this study align with recent qualitative research, which has highlighted the overall positive benefits of MC use for a variety of health conditions [58].

Similar positive and helpful effects have been documented in the literature, highlighting the potential benefits of MC for addressing various issues affecting patients with cancer [10]. Several studies have been published in recent years investigating the causal relationship between MC use and Quality of Life (QoL). Goldenberg et al. conducted a systematic review on recreational cannabis use and QoL, revealing that individuals who heavily used cannabis tended to have lower QoL [59]. Further recent studies have demonstrated beneficial short-term effects of MC on QoL [60]. Tait et al. [61]found that MC led to significant improvements in QoL, fatigue, pain, anxiety, and depression within the first three months of treatment, particularly among patients with pre-existing conditions. The study is ongoing to assess long-term effects over twelve months. Arkell et al. [62] noted that while adverse effects of MC were reported, they were rarely severe, and patients experienced sustained improvements in QoL across multiple domains. Olsson et al. [63] further confirmed significant improvements in QoL, anxiety, and sleep quality, with cannabis-based medicinal products proving generally well-tolerated. These findings suggest that MC is associated with sustained QoL benefits, but further research is required to refine clinical guidelines and ensure patient safety.

Another interesting discovery is that regarding positive experiences with society at large, some participants reported a noticeable improvement in public acceptance in recent years. Some had begun using MC before its legalization, requiring special approval for its use. However, the findings align with Reid’s interpretation: Assertions of normalization may be premature, as there is no evidence that stigma has entirely disappeared [29]. Participants emphasized that societal stigma remains a persistent issue for MC users. Several noted that older generations, in particular, continue to view cannabis primarily as a dangerous drug, a perspective shaped by historical prohibition policies. Moreover, some participants expressed concern that negative stereotypes about MC users’ educational backgrounds contribute to their marginalization.

The negative image of cannabis within communities could also stem from ignorance. Participants attribute these stereotypes and prejudices to a lack of information and knowledge. One participant specifically remarked that societal prejudices often outweigh scientific evidence in shaping public opinion.

All participants had at least one family member or friend who fully supported or at least accepted their use of MC. Many received unwavering support from family and close friends. Nearly all interviewees underscored the importance of such support, which serves as a potent protective factor against depression and contributes to enhancing positive effects [64]. However, some participants reported experiencing rejection, strained relationships, or even loss of contact with family members due to their MC use. One participant described that his brother, due to his profession, severed ties with him. Another noted that while his family tolerated his treatment, they still favoured alternative therapies. To navigate these challenges, several participants chose to disclose their MC use only to close confidants, viewing discretion as a safeguard against societal stigma.

Self-stigmatization emerged as a recurring issue, particularly in the early stages of treatment. One participant described how his fears of dependency were exacerbated by personal experiences with family members who had struggled with addiction. Concerns about addiction were a common theme among participants, with some questioning whether their use of MC was entirely socially accepted. Several participants also highlighted difficulties in dealing with law enforcement, citing a lack of police training in distinguishing between medical and recreational cannabis use.

The bureaucratic barriers to securing health insurance coverage for MC were a significant challenge repeatedly raised by participants. The complexity of the application process and frequent denials of reimbursement were cited as major obstacles. Further research should explore this issue in greater depth.

While a considerable number of participants express openness about their MC use, with some even proudly identifying as “cannabis users”, others remain apprehensive and choose to keep it private. One participant openly acknowledged that if better therapy options were available, they would readily opt for them. Though the main reasons for these differing opinions remain unclear, several factors may be assumed. Repeatedly mentioned was the concern about the smell of MC affecting others, prompting many to prefer taking their medication at home. Only a few interviewees seek secluded areas to inhale their medicine. A recent Australian study found that patients often explore alternative MC administration methods, such as capsules, to mitigate smell or avoid feeling excessively “high” [38]. These reported preferences and attitudes among MC users could be attributed to what Scambler termed “felt stigma” as opposed to “enacted stigma” [65]. While the latter denotes overt stigmatization, the former encompasses both a sense of shame and a corresponding fear of encountering stigma.

In this study, the interaction between patients and healthcare professionals emerged as a significant theme. Many participants in our study expressed dissatisfaction with their interactions with medical professionals, particularly in relation to the acceptance of MC. A recurring issue was the breakdown in communication between doctors and patients, especially once the patients’ use of MC became known. For some participants, this shift in treatment was accompanied by negative changes in how they were perceived by their healthcare providers. Despite some physicians being supportive, there was a general sentiment that more education and knowledge on MC were necessary. Participants emphasized that healthcare professionals should at least be open to referring them to colleagues who are more knowledgeable about cannabis therapy. This lack of understanding and openness from many healthcare providers contributed to feelings of alienation and dissatisfaction among MC users, illustrating the crucial role medical professionals play in supporting patient well-being.

Finding the “right” medical professional proves challenging, as few physicians and pharmacists are well-versed in MC. Nonetheless, some participants were fortunate not to have to search extensively, recognizing this as more of an exception than the norm. The role of medical professionals is pivotal, as patients’ perception of their competence and understanding can significantly impact the patient-provider interaction [66]. This positive correlation was evident in the present study as well. Understanding and openness exhibited by health professionals contributed to patient satisfaction and well-being. However, many interviewees did not share this “luck”. The majority experienced dissatisfaction due to breakdowns in doctor-patient communication [67]. Some reported that once their cannabis therapy became known, they perceived a shift in how they were treated by medical professionals.

A recurrent theme was the lack of acceptance of MC among healthcare providers. Many participants stressed the need for increased education and research on cannabis therapy. While some physicians demonstrated that their lack of experience did not prevent them from supporting patients, participants argued that, at a minimum, doctors should be willing to refer them to more knowledgeable colleagues.

The issue of inadequate training and knowledge among healthcare professionals regarding MC was also adressed. Tsampoula et al. [68] emphasized the importance of targeted training for healthcare professionals in developing personalized treatment plans and optimizing patient outcomes. Most of our intervewees stressed the need for increased education and research to ensure that cannabis therapy can be appropriately integrated into treatment plans. The lack of familiarity with MC, in many cases, led to unintentional stigmatization by healthcare providers, with some even rejecting it as a treatment option altogether. This knowledge gap resulted in a barrier to the adoption of MC as a viable treatment, limiting its accessibility for those who could potentially benefit from it. Participants strongly advocated for greater education for healthcare providers, asserting that lack of understanding should not justify the stigmatization or rejection of MC.

In this study, MC users detailed various challenges they encountered with healthcare professionals, expressing unfavourable experiences. On average, all participants cited at least one negative interaction. Throughout the results section, instances of inequality were noted, such as instances of denied medical examinations. It’s crucial to approach these observations critically, recognizing that while we grasp the patient’s perspective, the full context remains unclear. This qualitative inquiry resonates with the findings of Rønne et al. [69], which reveal a lack of clinical knowledge about MC among physicians.

In this context, stigma appears intertwined with knowledge gaps, as ignorance can breed potential bias or discrimination. Nearly all study participants advocated for greater education among healthcare professionals, asserting that lack of familiarity should not serve as grounds for wholesale stigmatization of MC. As noted by Mercurio et al., the failure of healthcare providers to furnish adequate knowledge regarding MC use acts as a barrier to its adoption as a treatment option [25]. These findings align with mine, as many participants observed that, for numerous medical practitioners, cannabis isn’t even considered a therapeutic option. Exploring the perspectives of healthcare professionals in greater depth would undoubtedly be enlightening. At present, however, any definitive conclusions would be premature. Nonetheless, we can acknowledge the existence of the issue, given the numerous first-hand experiences recounted by participants.

### Study limitations

The present study has several limitations that warrant consideration.

Firstly, there was an imbalance in the regional representation of the interviewees, with 12 out of 15 participants hailing from NRW. This may limit the generalizability of the findings to the entire federal territory of Germany, highlighting the need for further studies with a more diverse geographical distribution.

Secondly, recruiting participants under the age of 22 proved challenging, potentially skewing the results and limiting insight into the views of this age group. Similarly, there was a limitation in recruiting participants over the age of 60, which may have impacted the comprehensiveness of the findings across different age demographics.

Furthermore, while this study highlights key challenges faced by MC users in their interactions with healthcare professionals, the subjective nature of the self-reported data presents a limitation. Participants may have been influenced by social desirability bias, adjusting their responses to align with what they perceived as the study’s goals or the socially accepted views on cannabis use.

Finally, the study also relied on participants’ personal accounts of their experiences, which, while rich in detail, are not objective measures of the broader effectiveness of MC as a treatment. We were unable to assess the direct clinical outcomes or quantify the exact improvements in quality of life attributed to cannabis use.

These limitations should be taken into account when interpreting the findings of this study, and future research efforts should aim to address these shortcomings for a more comprehensive understanding of the topic [70].

## Conclusion

This study provides critical first-hand insights into the experiences of MC users, offering a nuanced understanding of both the benefits and challenges associated with cannabis therapy. By drawing on perspectives from individuals with chronic illnesses, including cancer, it highlights the significant improvements in quality of life reported by many participants. Positive effects, such as reduced pain, increased energy levels, and overall well-being, were frequently mentioned, underscoring the therapeutic potential of MC. Additionally, the study reflects the growing societal acceptance of MC, with some participants noting a shift towards greater public openness in recent years.

A key strength of this study lies in its detailed exploration of the patient-healthcare provider dynamic, an often-overlooked aspect of MC research. While some participants reported supportive and understanding doctors, many described persistent challenges in accessing appropriate medical care. The findings highlight a significant knowledge gap among healthcare professionals, reinforcing the need for improved education and training in cannabis therapy. A more informed and receptive medical community could not only enhance provider-patient relationships but also help reduce the stigma that continues to surround MC use.

Beyond individual patient experiences, this study offers a broader perspective on the structural and societal barriers faced by MC users. Despite its recognised benefits, MC is still not fully accepted as a legitimate treatment option within the medical establishment. The bureaucratic complexities associated with obtaining prescriptions and securing reimbursement further exacerbate these difficulties. Additionally, while participants generally found support among close friends and family, several reported strained relationships due to lingering misconceptions about MC. These findings suggest that stigma—both societal and self-imposed—remains a central issue, shaping the way MC users navigate their treatment and social interactions.

Nevertheless, the results indicate that positive experiences with MC extend beyond its medical effects. For many participants, successful symptom management led to improvements in employment, financial stability, and overall mental health. These aspects reinforce the broader value of MC, not only as a medical intervention but as a means of enhancing daily functioning and social participation. However, the persistent stigma and regulatory hurdles underscore the urgent need for policy reforms aimed at improving access, simplifying administrative processes, and fostering greater acceptance within the healthcare sector.

In light of these insights, future efforts should prioritise education at multiple levels, targeting healthcare professionals, policymakers, and the wider public. Addressing both enacted and felt stigma requires a multifaceted approach that combines evidence-based information with meaningful structural changes. Additionally, further research is essential to refine clinical guidelines, ensure patient safety, and integrate MC more effectively into mainstream medical practice. Ultimately, this study highlights the fundamental right of patients to receive equitable treatment, reinforcing the need for MC to be approached with the same professionalism and legitimacy as any other therapeutic option.

## Supplementary Information

Additional File 1. Interviews in the original language

Additional File 2. Information on the COREQ standard

## Data Availability

The datasets generated and analysed during the current study are not publicly available to ensure privacy protection for the participants. However, they can be made available from the corresponding author upon reasonable request. Please contact Velimir Borojevic at velimir.borojevic@gmail.com for inquiries regarding access to the datasets.

## Footnotes

1 The Association of German Cannabis Patients e.V. is a non-profit organization that sees itself as an advocacy group for people who can benefit from therapy with cannabinoids. (https://www.lobbyregister.bundestag.de/suche/R002445)

2 https://www.uniklinik-duesseldorf.de/patienten-besucher/klinikeninstitutezentren/institut-fuer-geschichte-theorie-und-ethik-der-medizin/forschung/ags-1/oral-history

3 The original wording of the interviews in German can be found in Additional file 1.
