# Supplementary material for: Detecting and understanding potential stigma among medical cannabis users in Germany
Source: BMC Public Health. 2025 Mar 5;25:874. doi: 10.1186/s12889-025-22084-w (PMC11884029; doi:10.1186/s12889-025-22084-w)
Supplement: Supplementary file 1 — Additional File 1. Interviews in the original language [file 12889_2025_22084_MOESM1_ESM.docx]

Text of the interviews English – original: German (order as appearing within the article)

Results / Society

*“At work they told me: No, you can't take your medicine here and please don't take it before you go on duty, because they said that you are under the influence of drugs and not focused” (p6). -* „Auf der Arbeit wurde dann gesagt zu mir Ja, nee, also hier können Sie Ihre Medizin nicht nehmen und lassen Sie es bitte auch vor dem Dienst, weil die dann gesagt haben, sie stehen unter Drogeneinfluss und sind nicht aufnahmefähig.“ (p6)

- *“Cannabis users are not dangerous, but someone who uses cannabis is a cannabis user and not a serious criminal. Yes, in society this image should also be reconsidered, that these stereotypes were made by people who wanted to denounce others. I don't need to explain the story of prohibition to you. You certainly know yourself that this has racist and economic backgrounds” (p12).-* „Cannabiskonsumenten sind nicht gefährlich, sondern jemand der Cannabis konsumiert ist halt ein Cannabiskonsumenten und kein schwer Krimineller. Ja, in der Gesellschaft sollte dieses Bild auch mal überdacht werden, dass diese Klischees von Menschen gemacht wurden, die andere denunzieren wollten. Die Geschichte (vom vom) von der Prohibition brauche ich ja Ihnen nicht erklären. Sie wissen ja bestimmt auch selber, dass das rassistische Hintergründe hatte und wirtschaftliche.“ (p12)
- *“I think even if you have not had any contact with cannabis at some point in your life, then you have grown up very conservatively. Then, is the gateway drug, so to speak. And that's what I was told when I was a teenager, in my case, by my parents“ (p5). -* „Ich glaube, auch wenn man keine Berührung mit Cannabis hatte irgendwann in seinem Leben, dann ist man in dieser Sache auch glaube ich sehr und sehr konservativ aufgewachsen (ist), dann ist das quasi die Einstiegsdroge. Und so wurde mir das auch mal vermittelt, als als Jugendlicher in meinem Fall, von meinen Eltern.“ (p5)
- *“[…] Even people with higher education, because it is always assumed that these who smoke pot are only the losers in education, which of course is not true at all “ (p9). -* „[…] Auch Menschen mit höheren Bildungsabschlüssen, weil man ja immer unterstellt, das sind nur die Bildungsversager, die kiffen oder so was natürlich überhaupt nicht stimmt.“ (p9)
- *“First of all, because of course this stoner image, which has been painted for decades, is still quite present “ (p4). -* „Also mal, weil natürlich dieses Bild von diesem Kiffer, was jetzt jahrzehntelang gezeichnet wurde, immer noch recht präsent ist“ (p4)
- *“Because you see it again and again in media reports, even when it comes to medical cannabis, that there is ALWAYS a super negative undertone [to put it very clearly]. Yes, you should be critical, you should. But you should be critical with every medication, with all opiates. You should be critical” (p1). –* „Denn man sieht das immer wieder in Medienberichten, auch wo es um medizinisches Cannabis geht, das IMMER ein super negativer Unterton darin herrscht[ganz deutlich gesagt]. Ja, man sollte kritisch sein, das sollte man. Aber bei jedem Medikament sollte man, bei allen Opiaten. Das sollte man.“ (p1)
- *“Um, to make it short Bob Marley equals Bob Marley T-Shirt, dreadlocks and somehow a bag[joint]. And this is the bad thing that somehow many patients also add up to this cliché...“ (p11).-* „Ähm, um es kurz zu machen, Bob Marley zu Bob Marley T-Shirt, Dreadlocks und irgendwie ne Tüte bis irgendwie zum Dings. Und es ist ja auch das Schlimme, dass irgendwie viele Patienten dieses Klischee ja auch füttern.“ (p11)
- *“[...] that they are also normal people who have hobbies, who have a family and whose hobbies are not related to cannabis from morning to night, but who just live a normal life and who should be let live. And I think that's what people will then learn“ (p9). -* „[…], dass es auch ganz normale Menschen sind, die Hobbys haben, die eine Familie haben und deren Hobby nicht von morgens bis abends mit Cannabis zusammenhängt, sondern die ein ganz normales Leben einfach nur leben und die man eben auch leben lassen sollte. Und ich denke, das werden die Leute dann lernen.“ (p9)
- *“I think you can also sometimes assess patients a little bit in terms of whether this is a patient who is now very open to, let´s say pharmaceuticals, psychotropic drugs, these classic things that are also used very successfully. I don't want to make it look bad or diverting attention away [from alternative approaches]. That's also the idea of homeopathy, things like that. Yes, maybe you have to believe in it. There is no scientific evidence. And yet there are very many people who say it helps them [….] We have six children and I don't want anything to happen about it, because it is not yet recognized in society. My wife is also a cannabis patient and we are suddenly called a ‘stoner family’. What's going on with them? We'll have to take a look. Things like that [meant: it is concerning and necessitates a closer examination of such matter]“ (p14). -* „Ich glaube, man kann Patienten schon auch manchmal in Richtungen ein bisschen einschätzen, ob das ein Patient ist, der jetzt sehr offen ist für, ich sage mal Medikamente, Psychopharmaka, diese klassischen Dinge, die ja auch sehr erfolgreich eingesetzt werden. Ich will da nichts schlecht machen oder aber zu anderen Dingen eher neigen. Das ist ja auch die Frage Homöopathie, solche Dinge. Ja, man muss wohl dran glauben. Wissenschaftliche Belege gibt es nicht. Und dennoch gibt es sehr viele Menschen, die sagen es hilft mir[…..]Wir haben sechs Kinder und ich möchte auf keinen Fall, dass darüber irgendetwas geschieht, weil es eben in der Gesellschaft noch nicht so anerkannt ist. Meine Frau ist ja auch Cannabis Patientin und dann plötzlich heißt (aus) Kiffer- Familie: ‚Was ist bei denen los? Da müssen wir mal nachschauen.‘ Solche Dinge.“ (p14)

Results / Family

- *“[...] because my brother has completely broken off contact with me because of this. So now I had nothing to do with him for four years [....] he studies administration of justice[...] he meant, yes, as a cannabis patient I have a bad influence on him and I am quite dangerous for his profession. (But), because as a judicial officer he is not allowed to do such things and should not have anything to do with such people. That is really unbelievable” (p10). -* „[…], weil meinen Bruder deswegen den Kontakt zu mir komplett abgebrochen hat. Also habe ich jetzt seit vier Jahren überhaupt nichts(mit ihm?) zu tun[….] er studiert Rechtspflege[…].er hat gemeint, ja, als Cannabis Patient bin ich ein schlechter Einfluss für ihn und ich bin ganz gefährlich für seinen Beruf. Aber weil weil er ja als Rechtspfleger so was gar nicht darf und auch nicht mit solchen Leuten zu tun haben sollte. Das ist wirklich unglaublich.“ (p10)
- *“My family not at all. So, I've always left out completely at family celebrations. My parents eventually accepted it, because I got the costs covered, but they kept telling me to stop, you'll find something better. Another way [alternative treatment] to deal with the disease. And every time I went to visit my parents, I didn't take my medicine because [...] I would have had to go off the property every time to be able to take it. And which actually then puts me back in a position where I'm at risk again, because I'm in the public domain” (p6). -* „Meine Familie überhaupt nicht. Also ich habe das auch immer auf Familienfeiern komplett weg (sein?) gelassen. Meine Eltern haben das dann zwar akzeptiert, weil ich die Kostenübernahme bekommen habe, aber waren immer dabei mir zu sagen „So hört auf, du findest doch was Besseres“. Eine andere Methode mit der Krankheit umzugehen. Und jedes Mal, wenn ich meine Eltern besucht habe, habe ich meine Medizin dann auch nicht eigenommen, weil[…] Ich hätte dann immer vom Grundstück runter gehen müssen, damit ich das nehmen darf. Und was mich dann eigentlich wieder eine Position, wo ich dann wieder im Risiko bin, weil ich im öffentlichen Bereich stehe.“ (p6)
- *“Whereby openly dealing with it doesn`t mean that everyone knows. Well, if somebody knows, it`s our closest relatives and friends, from whom we assume that they are also, well, let`s say, trustworthy enough” (p14). -* „Wobei das offen umgehen ist nicht so, dass das jeder weiß. Also wirklich, (wenn?)dann unsere engsten Verwandten und Freunde, von denen wir davon ausgehen, dass die eben auch, ich sage mal ja, vertrauenswürdig genug sind.“ (p14)
- *“[…] we don't have to shout it from the rooftops” (p2). -* „[…] wir müssen es ja nicht an die große Glocke hängen“ (p2)

## *Results /* Personal-experience (Self-experience)

- *“So, I'm now convinced about the subject of cannabis[...] would never have thought, I would be anyway, I would have, you don't see it on me, I don't have red eyes, nor that I somehow think ‘the world is so beautiful’. No, it makes me spring into action. I can, it(cannabis) can do something. I can go out with friends. It gives a quality of life back! [...] that this plant is greatly underestimated [....] I have gained nine kilo(grams) because of it, I am able to go back to work, I'm partially employed [managed to go back to work on a. partial basis]” (p2). -* „Also ich bin überzeugt über (von dem?) Cannabis Thema mittlerweile[…]hätte es niemals gedacht, ich wäre trotzdem, ich wäre, (man hätte es mi?), man sieht es mir nicht an, ich habe weder rote Augen, noch dass ich irgendwie denke, die ´Welt ist so wunderschön´. Nein, ich werde dadurch aktiv. Ich kann, er kann was machen. Ich kann mit Freunden ausgehen. Es gibt eine Lebensqualität zurück! ...dass diese Pflanze total unterschätzt (wird?) [….] Ich habe neun Kilo dadurch zugenommen, ich kann wieder arbeiten, ich bin teilerwerbstätig.“ (p2)
- *“To tell you honestly, quite frankly, If I did not have to take it from tomorrow on, I would be the happiest person in the world” (p3). -* „Sage Ihnen ganz ehrlich ganz offen müsste ich es ab morgen nicht mehr nehmen, ich war der glücklichste Mensch der Welt.“ (p3)
- *“The sad thing is that sometimes I myself still have this stigma in my head and then tend to abstain from medication in a situation where it is actually appropriate in order to avoid possible conflicts[...] You are directly labelled as someone of a category where you actually don't belong and then they can't believe that either” (p7). -* „Das Traurige ist, dass ich teilweise noch immer im Kopf selber dieses Stigma habe und dann eher auf eine Medikation in einer Situation verzichte, wo es eigentlich angemessen ist, um möglichen Konflikten aus dem Weg zu gehen[…] Man wird direkt abgestempelt in eine Kategorie, wo man eigentlich nicht hingehört und die das dann auch nicht glauben können.“ (p7)
- *“When I did not get it on medical prescription, I stopped several times. I was always afraid that I would be addicted. Okay, that was because of my father's alcohol addiction. That's where I am. As far as addiction is concerned, I was pretty warned and that's why I always stigmatized myself.” (p11). -* „Es war ja auch so als ich das nicht medizinisch verordnet bekommen habe, habe ich ja mehrmals aufgehört. Ich hatte ja immer Angst, ich wäre süchtig. Okay, das war durch die Alkoholsucht von meinem Vater. Da bin ich. Was irgendwie Sucht angeht, war ich ziemlich gewarnt und deshalb habe ich mich da immer selbst stigmatisiert.“ (p11)
- *“[...] is simply that I think that particularly for example police officers or similar I think should be better trained on medical cannabis” (p1).-* „[…] ist einfach, dass ich finde, dass gerade auch zum Beispiel Polizeibeamte oder ähnliche finde ich besser auf medizinischen Cannabis geschult werden müssten.“ (p1)
- *“[...] the health insurance company has to, because it is a drug available on prescription, simply pay for it” (p12). -* „[...] die Krankenkasse muss, weil es ein verschreibungspflichtiges Medikament ist, das auch einfach bezahlen“ (p12)
- *“The medical service of the health insurance is partly already a very big obstacle for people to become cannabis patients… “ (p6). -* „Der Medizinische Dienst der Krankenkasse ist ja teilweise schon ein sehr großes Hindernis für Leute um Cannabis Patient zu werden...“ (p6)

## *Results /* Medical professionals

- *“Ultimately, if you say somewhere front ´I am cannabis patient´, the person´s gaze already changes. I noticed that in the hospital as well. I was in the hospital last year with a stomach ulcer. I had such stomach pain and when they heard cannabis, they didn't want to examine me properly anymore “ (p3). -* „Letztendlich, wenn Sie schon irgendwo vorne sagen Ich bin Cannabis Patient, dass der Blick ändert sich schon von der Person. Ich habe das auch im Krankenhaus gemerkt. Ich war letztes Jahr im Krankenhaus wegen einem Magengeschwür. Ich hatte so Magenschmerzen und als sie gehört haben Cannabis, die wollten mich nicht mehr richtig untersuchen.“ (p3)
- *“But I still have... also often the feeling that many doctors simply reject it” (p1). -* „Aber ich habe immer noch…auch häufig das Gefühl, dass viele Ärzte sich einfach dagegen wehren.“ (p1)
- *“And that is also where the main need for education lies, because there are very very few doctors open to cannabis therapy” (p8). -* „Und da liegt auch die überwiegende Aufklärung(Notwendigkeit?), weil es sehr sehr wenig aufgeschlossene Ärzte gegenüber einer Cannabinoide Therapie gibt.“ (p8)
- *“Yes, you're always a bit, how should I say it, either the Guinea pig or the stupid stoner on the other side. Okay, so that has a lot to do with this pigeonhole that I was talking about earlier. It's very difficult for people to make it. That's why so many people deal with cannabis abuse, that is what you find on paper of some ADHD patient, just because the doctors directly pigeonhole the individual, although that was just a medical self-experiment. That is really difficult and really annoying for people who are not as I am” (p11). -* „Ja, man ist immer so ein bisschen, wie soll man sagen, einmal das Versuchskaninchen oder halt auf der anderen Seite der dumme Kiffer. Okay, also das spielt sehr viel mit dieser Schublade, von der ich vorhin schon sprach, mit rein. Die (es?)halt für die Leute sehr schwer machen. Deshalb haben so viele Leute auch ein Cannabis-Abusus, dass gerade bei ADHS in den Papieren stehen, weil halt die Ärzte die Leute direkt in diese Schublade stecken, obwohl da einfach nur ein medizinischer Selbstversuch vorlag. Das ist richtig schwierig und richtig nervig für die Menschen, die nicht so stringent sind wie ich.“ (p11)
