# Supplementary material for: Detecting and understanding potential stigma among medical cannabis users in Germany
Source: BMC Public Health. 2025 Mar 5;25:874. doi: 10.1186/s12889-025-22084-w (PMC11884029; doi:10.1186/s12889-025-22084-w)
Supplement: Supplementary file 2 — Additional File 2. Information on the COREQ standard [file 12889_2025_22084_MOESM2_ESM.docx]

**Supplementary Materials Table of Contents**

1. Consent Script
2. Consent To Participate in the Study and Data Processing
3. Interview Guide
4. Recruitment Script
5. COREQ Checklist
6. Data Sharing Statement

**1. Detecting and understanding potential stigma among medical cannabis users in Germany - Consent Script**

We are conducting interviews with individuals who use medical cannabis in Germany to better understand their experiences and whether they feel stigmatised due to their treatment. Since medical cannabis was legalised in 2017, its use has grown, but opinions on it are still mixed. Cannabis is both an illegal drug and a recognised medicine, there is still confusion among the public, doctors, and the police.

With these interviews, we want to understand how medical cannabis users feel about society’s views, whether they face stigma, and how they deal with it. We also hope to find ways to improve awareness and policies to better support patients and healthcare professionals. We expect interviews to take about 30-60 minutes depending on your experiences.

Participation is voluntary, and you can stop any time or skip any questions you don’t want to answer. We’ll also ask to audio-record the conversation so we can create a written transcript of the interview. You may request we stop recording at any time, and may request comments be removed from the transcription as well.

We’ll also collect some general demographic information from participants, but we won’t collect participants’ names or the specific hospitals in which they work. All data will be stored electronically in password protected electronic data files on encrypted devices that only IRB-approved team members will be able to access, and we’ll remove any information that identifies you from the transcript. We plan to publish what we learn from this study, but we won’t include anything that could identify individuals participating.

Do you have any questions or concerns?

[If none AND/OR questions or concerns about participating addressed]

Do you agree to participate in the study?

[If yes, agrees to participate. The confirmation of participation is explained again and signed.]

Do you have any concerns about audio recording?

[If none AND/OR questions and concerns about audio recording addressed]

And to confirm, do you agree to me audio-recording our interview?

[If yes]

I’ll start the recorder now.

[Start recording]

*Original in German:*

*Wir führen Interviews mit Personen, die in Deutschland medizinisches Cannabis nutzen, um ihre Erfahrungen besser zu verstehen und herauszufinden, ob sie sich aufgrund ihrer Behandlung stigmatisiert fühlen. Seit der Legalisierung von medizinischem Cannabis im Jahr 2017 hat die Nutzung zugenommen, doch die öffentliche Meinung bleibt zwiegespalten. Da Cannabis sowohl eine illegale Droge als auch ein anerkanntes Medikament ist, gibt es weiterhin Unsicherheiten und Missverständnisse – sowohl in der Gesellschaft als auch bei Ärzten und der Polizei.*

*Mit diesen Interviews möchten wir herausfinden, wie medizinische Cannabiskonsumenten die gesellschaftliche Wahrnehmung einschätzen, ob sie Stigmatisierung erfahren und wie sie damit umgehen. Zudem hoffen wir, Ansätze zu identifizieren, um das Bewusstsein für das Thema zu schärfen und politische Maßnahmen zu verbessern, die sowohl Patienten als auch medizinisches Fachpersonal unterstützen. Die Gespräche werden je nach Erfahrung etwa 30 bis 60 Minuten dauern.*

*Die Teilnahme ist freiwillig, und Sie können das Interview jederzeit abbrechen oder Fragen unbeantwortet lassen. Wir würden das Gespräch gerne aufzeichnen, um später eine schriftliche Transkription zu erstellen. Sie können die Aufzeichnung jederzeit unterbrechen oder verlangen, dass bestimmte Aussagen aus dem Transkript entfernt werden.*

*Zusätzlich erfassen wir einige allgemeine demografische Informationen, jedoch keine Namen oder Angaben zu spezifischen Krankenhäusern. Alle Daten werden elektronisch auf passwortgeschützten und verschlüsselten Geräten gespeichert, auf die nur autorisierte Mitglieder des Forschungsteams Zugriff haben. Persönlich identifizierbare Informationen werden aus den Transkriptionen entfernt. Die Ergebnisse der Studie werden veröffentlicht, jedoch ohne Angaben, die Rückschlüsse auf einzelne Teilnehmer ermöglichen.*

*Haben Sie Fragen oder Bedenken?*

*[Wenn keine Fragen und/oder Bedenken zur Teilnahme geklärt wurden:]*

*Sind Sie mit der Teilnahme an der Studie einverstanden?*

*[Wenn ja, wird die Teilnahmebestätigung erneut erläutert und unterzeichnet.]*

*Haben Sie Bedenken hinsichtlich der Audioaufzeichnung?*

*[Wenn keine Fragen und/oder Bedenken zur Audioaufzeichnung bestehen:]*

*Zur Bestätigung: Sind Sie damit einverstanden, dass unser Gespräch aufgezeichnet wird?*

*[Wenn ja:]*

*Ich starte die Aufnahme jetzt.*

*[Aufnahme starten]*

**2. Detecting and understanding potential stigma among medical cannabis users in Germany - Consent To Participate in the Study and Data Processing**

**Consent to Participate in the Study**

**Title:** *Detecting and understanding the potential stigma among medical cannabis users in Germany – A qualitative study*
**Institution:** Public Health Postgraduate Programme, Heinrich Heine University Düsseldorf
**Interviewer:** Velimir Borojevic

I, ____________________, hereby grant permission for the interview conducted as part of the above-mentioned Master's thesis to be recorded, scientifically analysed, and used in academic texts.

I voluntarily agree to participate in the study. I have received a clear and comprehensive oral explanation regarding the nature, significance, risks, and implications of the study. I had the opportunity to ask questions, all of which were satisfactorily answered. I understand that I may ask further questions at any time in the future. I have read and understood the study information. I have had sufficient time to decide whether to participate.

I consent to my interview data being collected in a pseudonymised form (i.e. coded without my name, address, initials, or similar identifiers), stored on data carriers, and analysed by the study’s principal investigator. Any sharing of data within scientific collaborations will occur exclusively in an anonymised form. Personal details will be stored separately in a secure database. Upon completion of the study, interview recordings will be retained for at least 10 years.

My participation and consent to the use of my data are voluntary. I may discontinue the interview at any time and decline further participation without any disadvantages.

If verbatim quotes from my interview are used in publications, I will receive the manuscript for review and have a two-week period to object to the publication of my statements.

I understand that I may withdraw my consent to participate in the study at any time, either orally or in writing, without providing reasons and without any negative consequences. However, I acknowledge that already published findings cannot be revoked, as the analysis and publication have already taken place. In such a case, the audio recordings and transcripts will be sent to me.

All study personnel are bound by medical or psychological confidentiality obligations and must uphold data protection regulations.

I have been explicitly assured that data protection regulations will be observed. I have received, read, and understood a copy of the study information and consent form.

**Consent to Data Processing**

1. I consent to my interview data being recorded and stored in pseudonymised form (i.e. coded without my name, address, initials, or similar identifiers) in either paper or electronic format in the archive of the Oral History Research Centre in Erfurt. If necessary, my anonymised data (i.e. without any reference to my identity) may be shared with the following researchers involved in the study: Dr. Chantal Marazia and PD Dr. Felicitas Söhner, both from the Institute for the History, Theory, and Ethics of Medicine, Heinrich Heine University Düsseldorf.
2. I have been informed that I may withdraw my consent for the recording, storage, and use of my data at any time. Additionally, I have the right to request the complete deletion of my data, provided it has not yet been included in the analysis. I understand that in the case of anonymised data storage, later deletion upon my request will no longer be possible.
3. I consent to my data being retained for at least 10 years after the conclusion or termination of the study. After this period, my personal data will be deleted unless legal or statutory retention requirements prevent this.

Location and Date: ____________________
Name of Participant: ____________________
Signature: ____________________

*Original in German:*

*Einwilligung in die Studienteilnahme*

*Thema: Detecting and understanding the potential stigma among medical cannabis users in Germany- A qualitative study*

*Institution: Weiterbildungsstudiengang Public Health, Heinrich-Heine-Universität Düsseldorf*

*Interviewer: Velimir Borojevic*

*Hiermit räume ich ,____________________, den im Rahmen der obengenannten Masterarbeit das Recht ein, das von mir gegebene Interview aufzuzeichnen, wissenschaftlich auszuwerten und in Texten zu verwenden.*

*Ich erkläre mich bereit, an der oben genannten Studie freiwillig teilzunehmen. Ich bin mündlich ausführlich und verständlich über Wesen, Bedeutung, Risiken und Tragweite der Studie aufgeklärt worden. In einem ausführlichen Beratungsgespräch hatte ich die Möglichkeit, Fragen zu stellen, die wiederum zufriedenstellend beantwortet wurden. Das Stellen weiterer Fragen ist auch zum späteren Zeitpunkt jederzeit möglich. Ich habe den Text der Studienaufklärung gelesen und verstanden. Ich hatte ausreichend Zeit, mich für oder gegen die Teilnahme an der Studie zu entscheiden.*

*Ich willige ein, dass meine studienbezogenen Interviewdaten pseudonymisiert (d.h. kodiert ohne Angabe von Name, Anschrift, Initialen oder Ähnlichem) erhoben, auf Datenträgern gespeichert und vom Auftraggeber der Studie ausgewertet werden. Eine Weitergabe an Dritte im Rahmen einer wissenschaftlichen Kooperation erfolgt ausschließlich in anonymisierter Form. Angaben zu meiner Person werden in einer getrennten Datenbank erfasst. Nach Abschluss der Studie werden die Interviewaufzeichnungen mind. 10 Jahre aufbewahrt.*

*Meine Teilnahme an der Erhebung und meine Zustimmung zur Verwendung der Daten sind freiwillig. Ich kann das Interview jederzeit abbrechen und weitere Interviews ablehnen, ohne dass mir dadurch irgendwelche Nachteile entstehen.*

*Falls wörtliche Zitate aus dem Interview in Veröffentlichungen verwendet werden, erhalte ich das Manuskript zur Einsicht und mir steht eine zweiwöchige Einspruchsfrist zur Publikation der von mir getätigten Aussagen zu.*

*Mir ist bekannt, dass ich jederzeit und ohne Angaben von Gründen gegenüber des Befragenden meine Einwilligung zur Teilnahme an der Studie zurückziehen kann (mündlich oder schriftlich), ohne dass mir daraus Nachteile entstehen. Bereits publizierte Arbeiten sind von der Rücknahme der Einwilligung naturgemäß nicht betroffen (da die Auswertung bereits erfolgt und publiziert ist). Audiodokument und Aufzeichnung werden mir dann zugesandt.*

*Alle an der Studie beteiligten Mitarbeitenden unterliegen der ärztlichen oder psychologischen Schweigepflicht und damit der Wahrung des Datengeheimnisses.*

*Die Einhaltung der datenschutzrechtlichen Vorschriften wurde mir ausdrücklich zugesichert. Ein Exemplar der Studieninformation und Einwilligungserklärung habe ich erhalten, gelesen und verstanden.*

*Einwilligung in den Datenschutz*

*1) Ich willige ein, dass im Rahmen dieser Studie meine studienbezogenen Interviewdaten in Papierform oder auf elektronischen Datenträger im Archiv der Forschungsstelle Oral History in Erfurt pseudonymisiert (d.h. verschlüsselt, ohne Angabe von Namen, Anschrift, Initialen oder Ähnlichem), aufgezeichnet und gespeichert werden. Soweit erforderlich, dürfen die erhobenen Daten anonymisiert (d.h. ohne jeglichen Hinweis auf meine Person) an die in Forschung beteiligten Wissenschaftler weitergegeben werden; dies sind: Frau Dr. Chantal Marazia und Frau PD Dr. Felicitas Söhner, beide Institut für Geschichte, Theorie und Ethik der Medizin, Heinrich Heine Universität Düsseldorf).*

*2) Ich bin darüber aufgeklärt worden, dass ich meine Einwilligung in die Aufzeichnung, Speicherung und Verwendung meiner Daten jederzeit widerrufen kann. Ich habe jedoch auch das Recht, eine vollständige Löschung meiner Daten zu verlangen, sofern diese noch nicht in die Auswertung eingegangen sind. Ich bin mir bewusst, dass im Falle einer anonymisierten Speicherung eine spätere Löschung auf meinen Wunsch hin nicht mehr möglich ist.*

*3) Ich willige ein, dass meine Daten nach Beendigung oder Abbruch der Studie mindestens 10 Jahre lang aufbewahrt werden. Danach werden meine personenbezogenen Daten gelöscht, soweit dem nicht gesetzliche, oder satzungsgemäße Aufbewahrungsfristen entgegenstehen.*

*___________________________________________________________________________*

*Ort und Datum Name der/des Studienteilnehmenden Unterschrift*

**2. Detecting and understanding potential stigma among medical cannabis users in Germany - Interview Guide**

Think about your past experiences with the use of medical cannabis. I would like to ask you about your perceptions and experiences in this context, with a particular focus on societal reactions and potential forms of stigma. Please note that this conversation is about your general experiences – you do not need to disclose any sensitive or personal information.

**1. Introductory Questions**

- Since when have you been using medical cannabis, and for which condition was it prescribed?
  - What medications did you try before?
  - Has medical cannabis allowed you to reduce or stop using other medications?
- How did you come to this decision?
  - Who suggested medical cannabis treatment to you? (Was it your own initiative, a doctor’s recommendation, or someone else’s suggestion?)
  - Was it difficult to find a doctor who would prescribe medical cannabis?
  - How would you assess the level of knowledge about medical cannabis within the healthcare system? Is it easy to find competent professionals?
- Have you ever faced issues with the police or administrative authorities (e.g. when obtaining a driving licence) due to your use of medical cannabis?
  - If so, how has this affected your daily life?

**2. Social Perception and Personal Experiences**

This part of the interview is semi-structured, meaning the order of the questions remains flexible, and follow-up questions are encouraged. The goal is to gain a deeper understanding of the participants' experiences, thoughts, and emotions regarding possible stigma.

- How do you think society perceives people who use medical cannabis?
- What kind of experiences have you had as a medical cannabis patient?
  - Can you describe any positive or negative experiences?
  - How did you react to these situations, and what did you take away from them?

**3. Reactions from Social Circles**

- How did your family and friends react when they found out about your treatment with medical cannabis?
- Do you believe that your social relationships have changed because of your medical cannabis use?
  - If so, in what way?
- Do you know anyone who has had negative social experiences due to their use of medical cannabis?

**4. Stigma and Self-Perception**

- Do you feel that you are treated differently from other patients because of your medical cannabis use?
- Have you ever felt ashamed or felt the need to hide your use of medical cannabis?
- Is it easier for you to talk openly about your use now compared to when you first started treatment?
- Do you think that societal acceptance of medical cannabis has increased in recent years?
  - If so, what makes you think so?

**5. Reflection and Areas for Improvement**

To conclude, participants will have the opportunity to share their overall thoughts, suggest areas for improvement, and provide feedback on the discussion.

- What changes would you like to see?
  - In terms of the healthcare system, society, health insurance providers, or political regulations?
- Is there anything I haven’t asked that you would like to share?

Original in German:

*Denken Sie an Ihre bisherigen Erfahrungen mit der Nutzung von medizinischem Cannabis. Ich möchte Sie zu Ihren Erlebnissen und Wahrnehmungen in diesem Zusammenhang befragen. Dabei interessieren uns insbesondere Ihre persönlichen Erfahrungen mit gesellschaftlichen Reaktionen und möglichen Formen der Stigmatisierung. Bitte beachten Sie, dass es in diesem Gespräch um Ihre allgemeinen Erfahrungen geht – Sie müssen keine sensiblen oder personenbezogenen Informationen preisgeben.*

***1. Einführende Fragen***

- *Seit wann nutzen Sie medizinisches Cannabis, und für welche Erkrankung wurde es Ihnen verschrieben?*
  - *Welche Medikamente haben Sie vorher ausprobiert?*
  - *Konnte durch die Behandlung mit Cannabis der Gebrauch anderer Medikamente reduziert oder beendet werden?*
- *Wie sind Sie zu dieser Entscheidung gekommen?*
  - *Wer hat Ihnen die Behandlung mit medizinischem Cannabis vorgeschlagen? (Eigeninitiative, Arzt, Dritte?)*
  - *War es schwierig, einen Arzt zu finden, der medizinisches Cannabis verschreibt?*
  - *Wie schätzen Sie das Wissen über medizinisches Cannabis im Gesundheitssystem ein? Ist es leicht, kompetente Ansprechpersonen zu finden?*
- *Haben Sie jemals Probleme mit der Polizei oder bei Behördengängen (z. B. beim Führerschein) aufgrund der Nutzung von medizinischem Cannabis gehabt?*
  - *Falls ja, wie hat sich das auf Ihr Leben ausgewirkt?*

***Hauptteil:*** *Der Hauptteil des Interviews ist teilstrukturiert. Die Reihenfolge der Fragen bleibt flexibel, und spontane Nachfragen sind erwünscht. In diesem Abschnitt soll ein tiefgehendes Verständnis für die Erfahrungen, Wahrnehmungen und Emotionen der Teilnehmenden im Zusammenhang mit möglicher Stigmatisierung gewonnen werden.*

***2. Gesellschaftliche Wahrnehmung und persönliche Erfahrungen***

- *Wie denken Sie, wird eine Person, die medizinisches Cannabis verwendet, von der Gesellschaft wahrgenommen?*
- *Welche Erfahrungen haben Sie aufgrund Ihres medizinischen Cannabiskonsums gemacht?*
  - *Können Sie positive oder negative Erlebnisse beschreiben?*
  - *Wie haben Sie auf diese Erfahrungen reagiert, und was haben Sie daraus gelernt?*

***3. Reaktionen des sozialen Umfelds***

- *Wie haben Familie und Freunde reagiert, als sie von Ihrer Behandlung mit medizinischem Cannabis erfahren haben?*
- *Glauben Sie, dass sich Ihre sozialen Beziehungen durch den Konsum von medizinischem Cannabis verändert haben?*
  - *Falls ja, in welcher Weise?*
- *Kennen Sie Menschen, die aufgrund der Nutzung von medizinischem Cannabis negative soziale Erfahrungen gemacht haben?*

***4. Stigmatisierung und Selbstwahrnehmung***

- *Haben Sie das Gefühl, aufgrund des medizinischen Cannabiskonsums anders behandelt zu werden als andere Patienten?*
- *Haben Sie sich jemals für Ihren medizinischen Cannabiskonsum geschämt oder das Bedürfnis verspürt, ihn zu verbergen?*
- *Fällt es Ihnen heute leichter, offen über Ihren Konsum zu sprechen als zu Beginn der Behandlung?*
- *Glauben Sie, dass die gesellschaftliche Akzeptanz von medizinischem Cannabis in den letzten Jahren gewachsen ist?*
  - *Falls ja, woran machen Sie das fest?*

***Abschluss des Interviews:*** *Zum Abschluss erhalten die Teilnehmer die Möglichkeit, ihre eigenen Gedanken zur Thematik zusammenzufassen, Verbesserungsvorschläge zu machen und ein abschließendes Feedback zum Gespräch zu geben.*

***5. Reflexion und Verbesserungspotenzial***

- *Welche Veränderungen würden Sie sich wünschen?*
  - *In Bezug auf das Gesundheitssystem, die Gesellschaft, Krankenkassen oder politische Regelungen?*
- *Gibt es etwas, das ich Sie nicht gefragt habe, das Ihnen aber wichtig ist zu erzählen?*

**3. Recruitment Script**

Dear Sir or Madam,

Since 2017, the German Bundestag has passed legislation allowing doctors to prescribe cannabis and regulating the reimbursement of treatment costs for medical purposes in Germany. Despite the increasing number of users, it remains unclear how the public (and even some professionals) perceive cannabis as a medicine. The question is how widely the use of medical cannabis is accepted in society.

We would like to understand your perspective on the overall situation—whether you believe that medical cannabis use is fully accepted in society, and if not, where improvements are needed (e.g. medical consultations, experiences with health insurance providers, or pharmacies). The findings should help determine whether better solutions are required, such as providing more information to the public and professionals.

For this purpose, I (Velimir Borojevic) will conduct interviews lasting approximately 30 minutes to a maximum of one hour. These interviews will take place online via video call. To ensure accuracy in capturing your responses, the interviews will be recorded.

As part of this study, we are required to collect your personal data. Each participant will be assigned a pseudonym. All study-related interview data will be stored and analysed solely under this pseudonym in a separate database—without any reference to names, initials, dates of birth, or similar identifying details. Pseudonymisation means that your name or other identifying characteristics will be replaced with a code, making it impossible or significantly more difficult to determine your identity. This "key" (linking your name or identifying characteristics to the code) will be stored separately from your other records or information. Access to the separately stored personal data will be limited to the study leadership and directly involved research staff.

Interview content may also be shared in anonymised form—meaning without any personal reference—with researchers involved in the study. These include Dr Chantal Marazia and PD Dr Felicitas Söhner, both from the Institute for the History, Theory, and Ethics of Medicine at Heinrich Heine University Düsseldorf.

Participating in this study poses no risks to you, though it is conceivable that the interview could trigger emotionally challenging personal memories. If this occurs, we will be happy to offer support and guidance.

Participation in the study is voluntary. If you decide to take part, please return the signed consent form. You may withdraw from the interview at any time and decline further participation without facing any disadvantages.

As a participant, you may exercise your rights under the EU General Data Protection Regulation (GDPR) at any time:

- The right to request information, including a free copy, about whether and which of your data is being processed (Article 15 GDPR).
- The right to request the correction or completion of your data (Article 16 GDPR).
- The right to request the deletion of your data in accordance with Article 17 GDPR.
- The right to request restrictions on data processing under Article 18 GDPR.
- The right to withdraw your consent at any time. This does not affect the lawfulness of processing carried out based on your consent before its withdrawal (Article 7(3) GDPR).
- The right to object to the future processing of your data in accordance with Article 21 GDPR.

If you believe your data is being processed unlawfully, you have the right to lodge a complaint with the following supervisory authority:

**State Commissioner for Data Protection and Freedom of Information North Rhine-Westphalia**
P.O. Box 20 04 44, 40102 Düsseldorf
Email: poststelle@ldi.nrw.de

Any previously collected data will be deleted upon request. However, anonymised data—meaning data without any personal reference—may continue to be used, as it will no longer be possible to trace it back to you. Please note that once data, samples, or recordings have been anonymised and incorporated into scientific analyses, they cannot be deleted or destroyed upon request. In line with scientific research regulations, all study data will be retained for at least ten years after the study concludes.

If you have any questions, concerns, or complaints regarding data processing and compliance with data protection regulations, please contact the study lead at velimir.borojevic@hhu.de.

As the interviews will be conducted online, no travel-related risks apply, and no additional insurance has been arranged for study participants.

I would be delighted if you chose to take part in this study.

You can contact me at any time via email at velimir.borojevic@hhu.de.

If you would like to learn more about the study, please let me know.

Thank you for your interest.

Velimir Borojevic
Principal Researcher and Co-Researchers

*Original in German:*

*Sehr geehrte Damen und Herren,*

*Seit 2017 hat der Bundestag Gesetz verabschiedet, das es Ärzten erlaubt, Cannabis zu verschrieben und die Erstattung der Kosten für die Therapie zu medizinischen Zwecken in Deutschland zu regeln. Trotz der zunehmenden Verbreitung der Nutzer scheint es, es ist noch nicht klar wie die Öffentlichkeit (und auch einige Fachleute) Cannabis als Medizin sehen. Die Frage ist, wie der Konsum von medizinischem Cannabis in der Gesellschaft akzeptiert ist.*

*Wir möchten herausfinden, wie Sie die gesamte Situation wahrnehmen, ob Sie denken, dass die Verwendung von medizinischem Cannabis in der Gesellschaft vollständig akzeptiert ist, und wenn nicht, wo Verbesserungen erforderlich sind (Arztbesuche, Erfahrungen mit Krankenversicherungen, Erfahrungen in Apotheken). Die Ergebnisse sollen zeigen, ob bessere Lösungen erforderlich sind (z.B. mehr Informationen für die Öffentlichkeit und Fachleute).*

*Dazu werde ich (Velimir Borojevic) Interviews mit einem Zeitaufwand etwa 30 Minuten bis max. 1 Stunde durchführen. Das Interview sollte Online geführt werden (Videotelefonie). Damit wir uns exakt an Ihre Antworten erinnern, wird das Interview aufgezeichnet.*

*In Rahmen dieser Studie ist erforderlich, Ihre Personendaten zu erfassen. Jeder Teilnehmerin/jedem Teilnehmer wird ein Pseudonym zugeordnet. Alle studienbezogenen Interviewdaten werden nur unter diesem Pseudonym in einer eigenen separaten Datenbank gespeichert (d.h. ohne Angabe von Namen, Initialen, Geburtstagdatum oder Ähnlichem) und ausgewertet. Pseudonymisierung bedeutet, dass Ihr Name oder andere Identifikationsmerkmale durch einen Code ersetzt werden, um die* [*Feststellung Ihrer Identität*](https://de.wikipedia.org/wiki/Identit%C3%A4tsfeststellung) *auszuschließen oder wesentlich zu erschweren. Dieser „Schlüssel“ (Verbindung zwischen Ihrem Namen bzw. Ihren Identifikationsmerkmalen und dem Code) wird getrennt von Ihren übrigen Befunden oder Informationen aufbewahrt. Auf die separat erfassten Personendaten der Studienteilnehmer können nur die Studienleitung und die unmittelbar mit der Studie befassten wissenschaftlichen Mitarbeiter zugreifen.*

*Interviewinhalte können darüber hinaus in anonymisierter Form, das heißt ohne jeglichen Personenbezug, mit den der Forschung beteiligten Forschende ausgetauscht werden, dies sind: Dr. Chantal Marazia und PD Dr. Felicitas Söhner, beide Institut für Geschichte, Theorie und Ethik der Medizin, Heinrich-Heine-Universität Düsseldorf.*

*Durch die Teilnahme an der Studie entstehen Ihnen keine Risiken, gleichwohl es denkbar ist, dass durch das Interview unter Umständen belastende persönliche Erinnerungen wachgerufen werden. In einem solchen Fall stehen wir Ihnen gern zur Beratung und Hilfe zur Verfügung.*

*Die Teilnahme an der Studie ist freiwillig. Wenn Sie sich für eine Teilnahme entschließen, geben Sie mir bitte die unterschriebene Einwilligungserklärung zurück. Sie können Interview jederzeit abbrechen und weitere Interviews ablehnen, ohne dass mir dadurch irgendwelche Nachteile entstehen.*

*Sie können als betroffene Person jederzeit die Ihnen durch die EU-DSGVO gewährten Rechte geltend machen:*

- *das Recht auf Auskunft inkl. kostenfreie Kopie, ob und welche Daten von Ihnen verarbeitet werden (Art. 15 EU-DSGVO),*
- *das Recht, die Berichtigung oder Vervollständigung der Sie betreffenden Daten zu verlangen (Art. 16 EU-DSGVO),*
- *das Recht auf Löschung der Sie betreffenden Daten nach Maßgabe des Art. 17 EU-DSGVO,*
- *das Recht, nach Maßgabe des Art. 18 EU-DSGVO eine Einschränkung der Verarbeitung der Daten zu verlangen,*
- *das Recht eine erteilte Einwilligung jederzeit zu widerrufen. Die Rechtmäßigkeit der aufgrund der Einwilligung bis zum Widerruf erfolgten Verarbeitung wird dadurch nicht berührt (Art. 7 Abs. 3 EU-DSGVO),*
- *das Recht auf Widerspruch gegen eine künftige Verarbeitung der Sie betreffenden Daten nach Maßgabe des Art. 21 EU-DSGVO.*

*Im Falle einer rechtswidrigen Datenverarbeitung haben Sie das Recht, sich bei folgender Aufsichtsbehörde zu beschweren:*

*Landesbeauftragte für Datenschutz und Informationsfreiheit Nordrhein-Westfalen*

*Postfach 20 04 44, 40102 Düsseldorf*

*E-Mail:* [*poststelle@ldi.nrw.de*](mailto:%20poststelle@ldi.nrw.de)

*Bereits erhobene Daten werden dann auf Ihren Wunsch hin gelöscht. Anonymisiert, d.h. ohne dass ein Personenrückschluss möglich ist, dürfen die Interviewdaten weiter genutzt werden, auch weil wir genau Ihre Angaben dann nicht mehr auffinden können. Wir weisen darauf hin, dass bereits anonymisierte Daten/Proben/Aufnahmen und Daten, die in wissenschaftliche Auswertungen eingeflossen sind, nicht mehr auf Wunsch gelöscht/vernichtet werden können****.*** *Nach Abschluss der Studie werden die Daten, wie für wissenschaftliche Studien vorgeschrieben, mind. 10 Jahre aufbewahrt.*

*Bei Anliegen, Fragen oder Beschwerden zur Datenverarbeitung und zur Einhaltung der datenschutzrechtlichen Anforderungen wenden Sie sich bitte zunächst an Studienleiter unter* [*velimir.borojevic@hhu.de*](mailto:velimir.borojevic@hhu.de)

*Da die Interviews online stattfinden, entstehen keine relevanten Risiken, beispielsweise durch Wege; eine separate Versicherung wurde für die Teilnehmenden dieser Studie nicht abgeschlossen.*

*Ich freue mich, wenn Sie sich zu einer Teilnahme an dieser Studie entschließen würden.*

*Sie können mich jederzeit per mail unter* [*velimir.borojevic@hhu.de*](mailto:velimir.borojevic@hhu.de) *kontaktieren.*

*Wenn Sie mehr über die Studie erfahren möchten, lassen Sie es mich bitte ebenfalls wissen.*

*Vielen Dank für Ihr Interesse,*

*Velimir Borojevic, Hauptforschender und Co-Forschende*

**3. Consolidated criteria for reporting qualitative studies (COREQ): 32-item checklist**

Developed from:

Tong A, Sainsbury P, Craig J. Consolidated criteria for reporting qualitative research (COREQ): a 32-item checklist for interviews and focus groups. *International Journal for Quality in Health Care*. 2007. Volume 19, Number 6: pp. 349 – 357

| **No. Item** | **Guide questions/description** | **Reported on Page #** |
| --- | --- | --- |
| **Domain 1: Research team and reﬂexivity** |  |  |
| *Personal Characteristics* |  |  |
| 1. Interviewer/facilitator | Which author/s conducted the interview or focus group? | 8 |
| 2. Credentials | What were the researcher’s credentials? E.g. PhD, MD | 9, title page |
| 3. Occupation | What was their occupation at the time of the study? | title page |
| 4. Gender | Was the researcher male or female? | Interviewer is a man |
| 5. Experience and training | What experience or training did the researcher have? | 9, title page |
| *Relationship with participants* |  |  |
| 6. Relationship established | Was a relationship established prior to study commencement? | Before this study, the two researchers had worked together. V.B. was in the field of "Public Health", F.S. is in the field of "History, Theory and Ethics of Medicine" at the same university. |
| 7. Participant knowledge of the interviewer | What did the participants know about the researcher? e.g. personal goals, reasons for doing the research | The purpose of the research was explained as part of the informed consent process (consent script included in Appendix). |
| 8. Interviewer characteristics | What characteristics were reported about the interviewer/facilitator? e.g. Bias, assumptions, reasons and interests in the research topic | As part of the recruitment and informed consent process, we described our interest in the topic and position |
| **Domain 2: study design** |  |  |
| *Theoretical framework* |  |  |
| 9. Methodological orientation and Theory | What methodological orientation was stated to underpin the study? e.g. grounded theory, discourse analysis, ethnography, phenomenology, content analysis | Pg. 9-12  Qualitative content analysis according to Mayring A category system was used for the evaluation. Its basis is to assign text passages to so-called categories. Categories have an abstract, classifying character and are intended to reflect the content of the respective text passages. The formation of categories was carried out using a mixed deductive-inductive process. |
| *Participant selection* |  |  |
| 10. Sampling | How were participants selected? e.g. purposive, convenience, consecutive, snowball | Pg. 7, 9-11  To assess the adequacy of the sample size, we relied on the information power criteria proposed by Malterud et al. We have described the sampling process in Study population – Recruitment and inclusion criteria |
| 11. Method of approach | How were participants approached? e.g. face-to-face, telephone, mail, email | Pg. 6, Abstract |
| 12. Sample size | How many participants were in the study? | Pg. 6, 8, Abstract, Table 1 |

| 13. Non-participation | How many people refused to participate or dropped out? Reasons? | Because we used snowball sampling (which included having participants directly pass along information about the study to colleagues) and used social media, we cannot say how many eligible participants may have received information about the study.  All participants who expressed interest eventually completed interviews. |
| --- | --- | --- |
| *Setting* |  |  |
| 14. Setting of data collection | Where was the data collected? e.g. home, clinic, workplace | Pg. 10 |
| 15. Presence of non-participants | Was anyone else present besides the participants and researchers? | No one else was present with the interviewer, we did not verify whether others were present with the participants |
| 16. Description of sample | What are the important characteristics of the sample? e.g. demographic data, date | Pg. 10, Table 1 |
| *Data collection* |  |  |
| 17. Interview guide | Were questions, prompts, guides provided by the authors? Was it pilot tested? | Pg. 10, electronical appendix, the interview guide was used across interviews |
| 18. Repeat interviews | Were repeat interviews carried out? If yes, how many? | NA |
| 19. Audio/visual recording | Did the research use audio or visual recording to collect the data? | Pg. 10 |
| 20. Field notes | Were ﬁeld notes made during and/or after the interview or focus group? | Interview summaries were written, Pg. 10. |
| 21. Duration | What was the duration of the interviews or focus group? | Pg. 10 |
| 22. Data saturation | Was data saturation discussed? | Pg. 7 |
| 23. Transcripts returned | Were transcripts returned to participants for comment and/or correction? | No |
| **Domain 3: analysis and ﬁndings** |  |  |
| *Data analysis* |  |  |
| 24. Number of data coders | How many data coders coded the data? | Pg. 7; VB constructed initial tables with review of raw data in tables by FS, review of reduced tables by the authors and the oral history research group |
| 25. Description of the coding tree | Did authors provide a description of the coding tree? | Pg. 13 |
| 26. Derivation of themes | Were themes identiﬁed in advance or derived from the data? | Specific topics were identified in advance, conceptualization within categories was derived from the data |
| 27. Software | What software, if applicable, was used to manage the data? | Pg. 2, 10, 11 |
| 28. Participant checking | Did participants provide feedback on the ﬁndings? | No |
| *Reporting* |  |  |
| 29. Quotations presented | Were participant quotations presented to illustrate the themes/ﬁndings? Was each quotation identiﬁed? e.g. participant number | Yes, Pg. 10-14 |
| 30. Data and ﬁndings consistent | Was there consistency between the data presented and the ﬁndings? | Yes |
| 31. Clarity of major themes | Were major themes clearly presented in the ﬁndings? | Yes, Pg. 10-14, Tables and Figures |
| 32. Clarity of minor themes | Is there a description of diverse cases or discussion of minor themes? | Variation within categories is described in pg. 10-14 |

**4. Data Sharing Statement**

Data available: No

Interview protocol explicitly specified protecting interview participants confidentiality, thus, in this study we also did not specify personal names, organization names or provider roles with quotations to avoid risk of identifying study participants.
